# Mental Health Profiles in Children and Adolescents with Visual Impairment: The Role of Syndromic Etiology and Vision Severity

**DOI:** 10.3390/medsci14050529

**Published:** 2026-08-28

**Authors:** Mara L. Cordeiro, Tiago S. Bara, Vanessa Furlin, Daniel Almeida do Valle, Perola G. Iankilevich, Carolina Prando

**Affiliations:** 1 Faculdades Pequeno Principe, Curitiba 80230-020, PR, Brazil; tiago.bara@pelepequenoprincipe.org.br (T.S.B.); daniel.valle@hpp.org.br (D.A.d.V.); perolagrup@gmail.com (P.G.I.); carolina.prando@hpp.org.br (C.P.); 2 Instituto de Pesquisa Pelé Pequeno Príncipe, Curitiba 80250-060, PR, Brazil; vanessa.furlin@pelepequenoprincipe.org.br; 3 Hospital Pequeno Principe, Curitiba 80250-060, PR, Brazil; 4 Department of Psychiatry and Biobehavioral Sciences, David Geffen School of Medicine, University of California Los Angeles, Los Angeles, CA 90095, USA

**Keywords:** visual impairment, Child Behavior Checklist, syndromic, severity of visual impairment, emotional and behavioral problems, children, adolescents

## Abstract

**Background:** Visual impairment is associated with mental health difficulties in children, yet emotional and behavioral profiles across different etiologies and levels of visual impairment remain poorly characterized. We investigated whether these outcomes were associated with syndromic etiology or visual impairment severity. **Methods:** In this cross-sectional study, parents of 69 children and adolescents aged 6–17 years with visual impairment completed the Child Behavior Checklist (CBCL/6–18). Participants were grouped by etiology (syndromic, *n* = 14; non-syndromic, *n* = 55) and by visual impairment severity (mild-to-moderate, *n* = 33; severe-to-profound, *n* = 36). Group differences were assessed using Mann–Whitney U tests with false discovery rate (FDR) correction, complemented by categorical analyses using scale-specific CBCL clinical thresholds. **Results:** No significant differences in emotional or behavioral symptoms were observed between the syndromic and non-syndromic groups, although the small syndromic subgroup limited statistical power. In contrast, children with mild-to-moderate visual impairment had significantly higher scores on the Anxious/Depressed and Somatic Complaints scales than those with severe-to-profound impairment, with both differences remaining significant after FDR correction. Categorical analyses also showed a higher prevalence of clinically significant Anxious/Depressed and Internalizing Problems in the mild-to-moderate group. **Conclusions:** Children with mild-to-moderate visual impairment exhibited higher Anxious/Depressed and Somatic Complaints scores than those with severe-to-profound impairment. These findings suggest that children with residual vision may represent an underrecognized subgroup at risk for emotional difficulties and support routine psychological screening across the spectrum of pediatric visual impairment.

## 1. Introduction

Visual impairment affects millions of children worldwide and can substantially influence their educational attainment, social functioning, and mental health [1]. Beyond its direct sensory consequences, childhood visual impairment may disrupt multiple domains of neurodevelopment, including emotional regulation, social interaction, and behavioral adjustment, as visual input plays a fundamental role in early social learning, communication, and environmental exploration.

Evidence linking pediatric visual impairment to emotional and behavioral difficulties has grown in recent years, although it remains far more limited than the corresponding adult literature [1]. A systematic review and meta-analysis reported significantly higher levels of anxiety and depression among children with visual impairment compared with their normally sighted peers [2]. Likewise, a recent US national survey found an increased prevalence of mental health difficulties in children with visual impairment [1]. The Child Behavior Checklist (CBCL; [3]) is one of the most widely used parent-report measures for assessing these emotional and behavioral problems [4], providing standardized measures of internalizing symptoms (e.g., anxiety and depression), externalizing behaviors (e.g., aggression and rule-breaking), and emotional and behavioral problems oriented toward the Diagnostic and Statistical Manual of Mental Disorders (DSM) [5].

Visual impairment in childhood is etiologically heterogeneous, encompassing isolated ocular disorders as well as conditions associated with genetic syndromes that involve broader neurodevelopmental alterations [6]. Children with neurogenetic syndromes often exhibit distinct behavioral and emotional profiles compared with those with non-syndromic developmental disabilities [7]. Similarly, a study comparing children with visual impairment with and without co-occurring intellectual disability found that intellectual disability exerted a stronger influence than visual impairment itself on the prevalence and pattern of emotional and behavioral problems [8]. Together, these findings suggest that the underlying etiology, rather than the sensory deficit alone, may account for a substantial proportion of psychiatric risk in this population.

In addition to etiology, the severity of visual impairment has been proposed as an important factor associated with psychological adjustment [9]. A classic review identified both the cause and the extent of visual loss as key factors influencing emotional and behavioral adaptation, while emphasizing that the available evidence was insufficient to establish consistent relationships because of methodological limitations and the scarcity of controlled studies [10]. Despite decades of research, the independent contribution of visual impairment severity to mental health outcomes remains incompletely understood.

Few studies have simultaneously examined the relative contributions of syndromic etiology and severity of visual impairment to emotional and behavioral outcomes within the same pediatric sample, as noted in a recent systematic review [9]. Moreover, evidence from Latin America and other low- and middle-income countries remains scarce. Therefore, this study examined emotional and behavioral profiles in a Brazilian sample of children and adolescents aged 6–17 years, comparing participants by etiology (syndromic vs. non-syndromic) and severity of visual impairment (mild-to-moderate vs. severe-to-profound). Based on prior evidence linking greater severity of sensory loss to poorer psychological adjustment [2,10], we hypothesized that emotional and behavioral problems would be greater in children with syndromic than non-syndromic visual impairment and in those with severe-to-profound than mild-to-moderate visual impairment.

## 2. Materials and Methods

### 2.1. Design and Participants

This cross-sectional study analyzed data collected as part of the “SABER E VER” project, a multidisciplinary neurodevelopmental research program conducted at the Pelé Pequeno Príncipe Research Institute in Curitiba, Paraná, Brazil. The project comprises three interconnected components: ophthalmological, genetic, and neuropsychological assessments. Participant recruitment and ophthalmological characterization were initiated within the Pediatric Ophthalmology Service, with genetic and neuropsychological assessments conducted within the broader multidisciplinary research protocol. The project integrates neuropediatricians, pediatric ophthalmologists, and neuropsychologists. Neuropediatricians established syndromic diagnoses, pediatric ophthalmologists established the diagnosis and severity of visual impairment, and neuropsychologists conducted neurodevelopmental and behavioral assessments. Participants were recruited from the pediatric ophthalmology outpatient clinics of Hospital Pequeno Príncipe, a tertiary/quaternary pediatric referral center in Curitiba, Paraná, Brazil.

**Figure 1:**
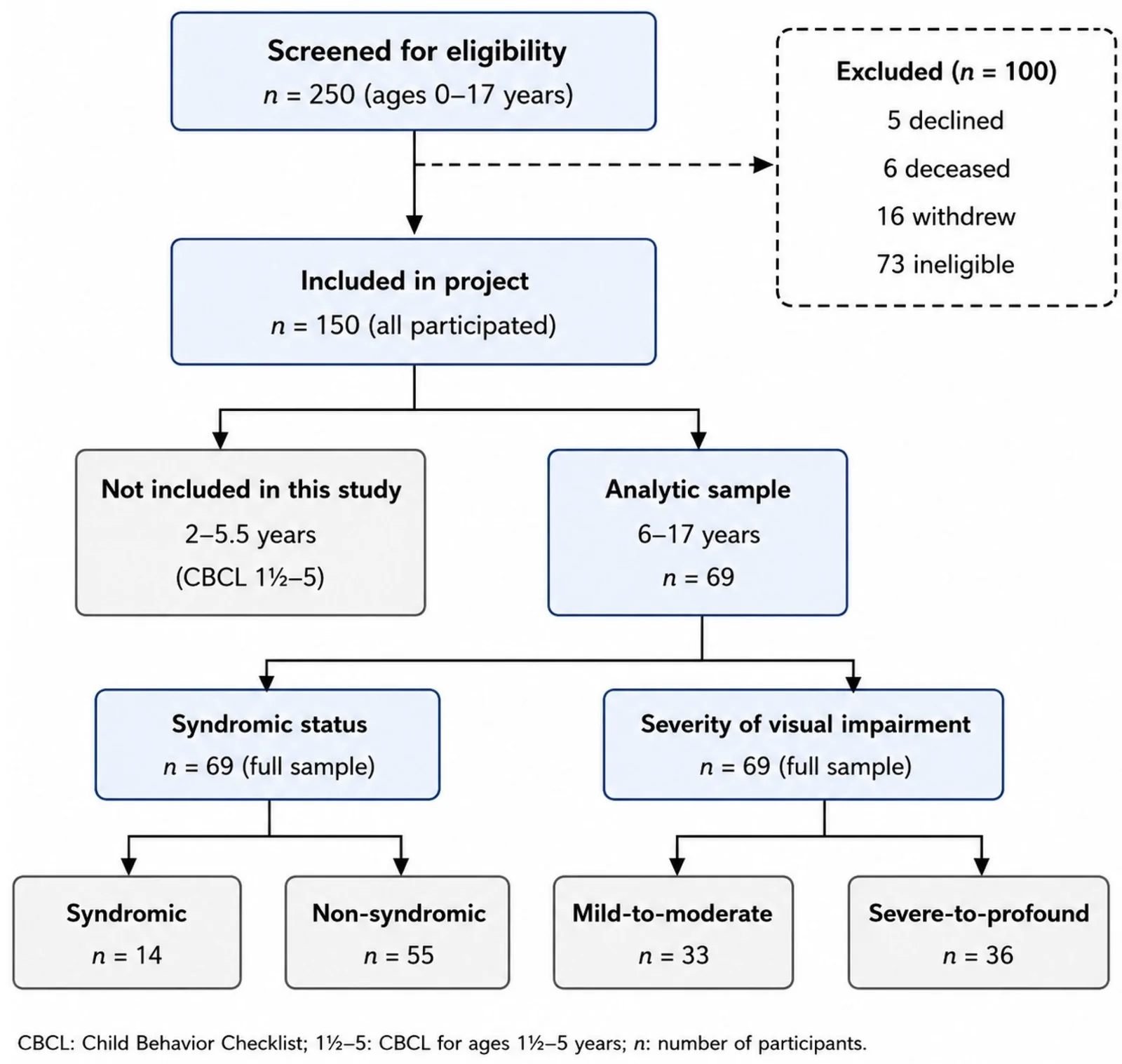
Participant flow diagram. Of 250 children and adolescents screened for eligibility, 100 were excluded (5 declined participation, 6 were deceased, 16 withdrew, and 73 did not meet the eligibility criteria), resulting in 150 participants enrolled in the SABER E VER neuropsychological project. Participants aged 2–5.5 years completed the CBCL/1½–5 and were not included in the present analysis. Participants aged 6–17 years who completed the CBCL/6–18 constituted the final analytic sample (*n* = 69). This sample was analyzed according to syndromic status (syndromic, *n* = 14; non-syndromic, *n* = 55) and visual impairment severity (mild-to-moderate, *n* = 33; severe-to-profound, *n* = 36). Arrows indicate the flow of participants through eligibility screening, project inclusion, and selection of the analytic sample and study subgroups.

Recruitment and eligibility information were obtained through medical record review and interviews with participants and their legal guardians. The overall SABER E VER project screened 250 children and adolescents aged 0–17 years for eligibility. Of these, 100 were excluded (5 declined participation, 6 were deceased, 16 withdrew, and 73 did not meet the eligibility criteria), resulting in 150 participants enrolled in the SABER E VER project. For the neuropsychological component, participants were stratified according to age-specific CBCL instruments. Participants younger than 2 years were not eligible for CBCL assessment. Children aged 2–5.5 years were assessed using the CBCL/1½–5 and were not included in the present analysis. The present study was restricted a priori to participants aged 6–17 years who completed the CBCL/6–18, yielding a final analytic sample of 69 participants (as detailed in Figure 1). A subset of this cohort has previously been described in a study examining cognitive and executive functioning [11].

For comparisons based on etiology, the final analytic sample comprised 69 participants, including 14 with syndromic and 55 with non-syndromic visual impairment. The syndromic subgroup encompassed a clinically heterogeneous range of genetic and congenital conditions, including conditions such as Bardet–Biedl syndrome (*n* = 2), Kabuki syndrome (*n* = 1), Joubert syndrome (*n* = 1), Coffin–Siris syndrome *(n* = 1), Goltz syndrome or focal dermal hypoplasia (*n* = 1), Cockayne syndrome (*n* = 1), Noonan syndrome (*n* = 1), and Behr syndrome with epilepsy (*n* = 1), as well as congenital malformation syndromes diagnosed on clinical and radiological grounds (e.g., myelomeningocele with hydrocephalus), without evidence of a monogenic disorder. Classification into the syndromic group was based on the established etiological diagnosis: for monogenic conditions, molecular criteria were applied, with diagnostic confirmation through identification of pathogenic or likely pathogenic variants related to the clinical presentation; for clinical syndromes without an established monogenic etiology, clinical and radiological criteria were used. Accordingly, the syndromic group included both participants with a molecularly confirmed diagnosis of a monogenic disorder and those with syndromes defined on clinical-radiological grounds without an identified monogenic etiology.

Written informed consent was obtained from legal guardians, and assent was obtained from participants when appropriate. The study was approved by the Brazilian National Research Ethics Committee (Comissão Nacional de Ética em Pesquisa—CONEP) (approval no. 4.195.697) on 6 August 2020. Recruitment could not begin immediately following approval owing to the COVID-19 pandemic, which suspended non-urgent in-person clinical research activities. Data collection subsequently occurred between 3 October 2022 and 10 October 2023.

### 2.2. Ophthalmological Assessment and Classification of Visual Impairment

Ophthalmological assessments were performed at the Pediatric Ophthalmology Service of Hospital Pequeno Príncipe by pediatric ophthalmologists using standardized clinical procedures and equipment. Visual impairment was determined based on best-corrected visual acuity (BCVA) in the better-seeing eye. Visual acuity was assessed using the Snellen chart when feasible; Teller Acuity Cards were used in children unable to complete conventional optotype-based visual acuity testing. All participants underwent a comprehensive ophthalmological examination, with additional ocular investigations performed when clinically indicated. The ophthalmological eligibility criterion for the SABER E VER project was a BCVA of 20/60 or worse in the better-seeing eye with optimal optical correction. Visual impairment severity was classified according to BCVA in the better-seeing eye, based on internationally recognized World Health Organization (WHO) visual impairment categories [12,13]. The WHO classification distinguishes moderate visual impairment, severe visual impairment, and blindness using progressively lower levels of distance visual acuity, with 20/200 representing the boundary between moderate and severe visual impairment and 20/400 the boundary between severe visual impairment and blindness.

For the primary analyses, these categories were consolidated into two clinically relevant groups using 20/200 as the threshold separating moderate from severe visual impairment: mild-to-moderate visual impairment (BCVA from 20/60 to 20/200; *n* = 33) and severe-to-profound visual impairment (BCVA worse than 20/200, including blindness and absence of light perception; *n* = 36). Visual acuity was dichotomized rather than analyzed as a continuous variable to align with clinically actionable categories used in rehabilitation and educational planning and to preserve statistical power given the sample size; this grouping was used to preserve clinically meaningful differences in residual visual function while maintaining adequate group sizes for statistical comparison. An exploratory correlation using the full ordinal severity classification is presented in Section 3.3 to examine whether behavioral symptoms varied across the ordinal levels of visual impairment severity. For the secondary exploratory analysis examining whether behavioral symptoms followed an ordinal gradient across visual impairment severity, the full six-level classification used in the study was retained [12,13]: mild visual impairment (*n* = 1), moderate visual impairment (*n* = 32), severe visual impairment (*n* = 2), profound visual impairment (*n* = 4), near-total blindness (*n* = 21), and total blindness (*n* = 9).

### 2.3. Behavioral Assessment

Emotional and behavioral symptoms were assessed using the parent-report Child Behavior Checklist for Ages 6–18 (CBCL/6–18) [3]. Parents completed the Brazilian version of the CBCL/6–18, whose development, cross-cultural adaptation, and psychometric properties are described in detail elsewhere [14,15]; internal consistency and factor structure for the Brazilian CBCL/6–18 have been reported to closely parallel those of the original US version [15]. Although the Brazilian CBCL/6–18 has not been specifically validated in samples of children with visual impairment or heterogeneous syndromic/neurodevelopmental conditions, it remains among the most widely used cross-culturally validated instruments for assessing emotional and behavioral symptoms in pediatric populations, including in prior studies of this cohort [11]. The CBCL generates standardized T-scores (mean = 50, SD = 10) for eight empirically derived syndrome scales (Anxious/Depressed, Withdrawn/Depressed, Somatic Complaints, Social Problems, Thought Problems, Attention Problems, Rule-Breaking Behavior, and Aggressive Behavior), six DSM-oriented scales, and three composite indices (Internalizing Problems, Externalizing Problems, and Total Problems). Higher T-scores indicate greater emotional and behavioral symptom severity. Raw CBCL item scores were converted into standardized, age- and sex-normed T-scores using the Assessment Data Manager (ADM) software from the Achenbach System of Empirically Based Assessment (v.4.3.3 ASEBA-PC 2023) [3], which applies the normative scoring algorithms for the CBCL/6–18 to generate syndrome scale, DSM-oriented scale, and composite index T-scores from raw item ratings.

### 2.4. Statistical Analysis

Descriptive statistics are presented as medians and interquartile ranges (IQRs) for each CBCL scale, stratified by syndromic status and by severity of visual impairment. Owing to the modest and unequal group sizes and the non-normal distribution of CBCL T-scores, between-group comparisons were performed using the Mann–Whitney U test. Categorical comparisons, including sex distribution, visual impairment severity distribution, and clinical-range prevalence, were performed using Fisher’s exact test because of small expected cell counts in several subgroups. Effect sizes were estimated using the rank-biserial correlation (r), calculated as r = 1 − 2U/(n_1_ × n_2_). To account for multiple comparisons across the 17 CBCL scales evaluated in each of the two primary group comparisons, *p*-values were adjusted using the Benjamini–Hochberg false discovery rate (FDR) procedure. FDR-adjusted *p*-values < 0.05 were considered statistically significant. The comparisons according to syndromic status and visual impairment severity constituted the primary analyses. The categorical analyses based on clinical CBCL thresholds and the Spearman rank-order correlation using the ordinal visual impairment severity classification were conducted as secondary exploratory analyses. All statistical analyses were performed using IBM SPSS Statistics, version 23.0 (IBM Corp., Armonk, NY, USA); results were independently cross-checked using Python 3 (pandas and SciPy libraries) in Google Colab. No outcome or visual-impairment severity data were missing in the final analytic sample, and no data were imputed.

## 3. Results

Table 1 summarizes the demographic characteristics of the study sample for both group comparisons. No significant differences in age or sex distribution were detected between the syndromic and non-syndromic groups (age *p* = 0.601; sex *p* = 0.559) or between the mild-to-moderate and severe-to-profound visual impairment groups (age *p* = 0.832; sex *p* = 0.464). Additionally, the distribution of visual impairment severity did not differ significantly between the syndromic and non-syndromic groups (*p* = 0.171).

### 3.1. Symptoms in the Clinical Range Across the Full Sample

Before examining group-level comparisons, we characterized the proportion of participants scoring at or above the predefined thresholds for clinical concern on each CBCL scale. Following ASEBA guidelines [3], the threshold for clinical concern was defined as T ≥ 65 for broadband composite scales (Internalizing, Externalizing, and Total Problems) and T ≥ 70 for individual syndrome and DSM-oriented scales. Using these thresholds, 54 participants (78.3%) had at least one elevated scale, whereas 15 participants (21.7%) had no scale scores at or above these thresholds (Table 2).

The most frequently elevated scales were Internalizing Problems (47.8%), Total Problems (47.8%), Withdrawn/Depressed (43.5%), and Social Problems (40.6%). The least frequently elevated scales were DSM-oriented Somatic Problems (5.8%), Rule-Breaking Behavior (10.1%), Oppositional Defiant Problems (13.0%), and Conduct Problems (13.0%). The Internalizing Problems composite was elevated in 33 participants (47.8%) and the Externalizing Problems composite in 21 (30.4%). Fifteen participants (21.7%) had elevations on both composites, whereas 18 (26.1%) had an Internalizing Problems elevation only, and 6 (8.7%) had an Externalizing Problems elevation only.

### 3.2. Syndromic vs. Non-Syndromic CBCL Profiles

**Figure 2:**
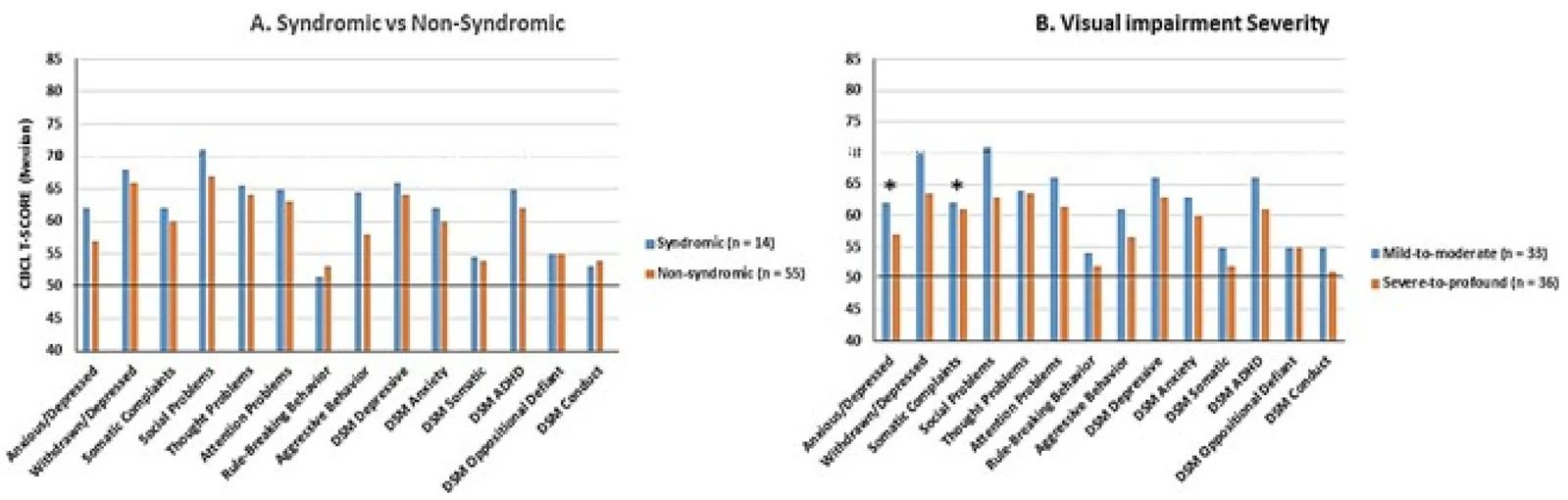
CBCL T-scores by syndromic status and visual impairment severity. Median CBCL T-scores according to syndromic status (**A**) and visual impairment severity (**B**). The solid horizontal line indicates the normative population mean (T = 50). A single universal clinical threshold is not displayed because clinical thresholds differ across CBCL scale types: T ≥ 65 for broadband composite scales and T ≥ 70 for syndrome and DSM-oriented scales. Asterisks (*) indicate the two scales with statistically significant between-group differences after false discovery rate (FDR) correction (FDR-adjusted *p* = 0.017): Anxious/Depressed and Somatic Complaints, both in panel (**B**). Sample sizes: syndromic, *n* = 14; non-syndromic, *n* = 55; mild-to-moderate, *n* = 33; severe-to-profound, *n* = 36.

No significant differences were observed between participants with syndromic (*n* = 14) and non-syndromic (*n* = 55) visual impairment on any CBCL syndrome, DSM-oriented, or composite scale, either before or after correction for multiple comparisons (all *p* > 0.32; Figure 2A). Median T-scores were comparable across groups for Internalizing Problems, Externalizing Problems, Total Problems, and all individual CBCL scales.

### 3.3. Visual Impairment Severity and CBCL Profiles

Emotional and behavioral symptom profiles differed according to visual impairment severity (Figure 2B). Participants with mild-to-moderate visual impairment (*n* = 33) had significantly higher scores than those with severe-to-profound visual impairment (*n* = 36) on the Anxious/Depressed syndrome scale (median T-score: 62.0 vs. 57.0; U = 172.0, *p* = 0.001; FDR-adjusted *p* = 0.017; r = −0.42, 95% CI [−0.60, −0.20]) and the Somatic Complaints syndrome scale (median T-score: 62.0 vs. 61.0; U = 330.5, *p* = 0.002; FDR-adjusted *p* = 0.017; r = −0.37, 95% CI [−0.57, −0.15]). Both differences remained statistically significant after FDR correction for multiple comparisons.

Four additional scales showed nominal between-group differences before correction for multiple comparisons, with higher scores in the mild-to-moderate group, but these differences did not remain statistically significant after FDR correction: Total Problems (*p* = 0.014; FDR-adjusted *p* = 0.075), Social Problems (*p* = 0.019; FDR-adjusted *p* = 0.075), DSM-oriented ADHD Problems (*p* = 0.022; FDR-adjusted *p* = 0.075), and DSM-oriented Conduct Problems (*p* = 0.046; FDR-adjusted *p* = 0.130). No significant differences were observed for continuous scores on the Internalizing Problems composite (*p* = 0.620) or Externalizing Problems composite (*p* = 0.626).

Secondary categorical analyses using the scale-specific ASEBA thresholds identified differences in the prevalence of clinically elevated scores according to visual impairment severity (Table 3). A higher proportion of participants with mild-to-moderate visual impairment scored at or above the threshold for clinical concern on the Anxious/Depressed scale compared with those with severe-to-profound impairment (27.3% vs. 5.6%, *p* = 0.022). Although continuous Internalizing Problems T-scores did not differ significantly between groups, the proportion of participants meeting the clinical threshold for Internalizing Problems was higher in the mild-to-moderate group (75.8% vs. 30.6%, *p* < 0.001). The proportion meeting the threshold for Withdrawn/Depressed was also higher in the mild-to-moderate group (60.6% vs. 27.8%, *p* = 0.005).

To explore whether emotional and behavioral symptoms varied along an ordinal gradient of visual impairment severity, Spearman’s rank-order correlations were conducted using the full six-level classification of visual impairment severity. No statistically significant correlations were observed between visual impairment severity and any CBCL scale. Specifically, no significant correlations were observed for the Anxious/Depressed syndrome scale (ρ = −0.027, *p* = 0.867) or the Internalizing Problems composite (ρ = 0.137, *p* = 0.350).

## 4. Discussion

This study examined emotional and behavioral profiles in Brazilian children and adolescents with visual impairment, comparing participants according to syndromic status and visual impairment severity. Three main findings emerged. First, emotional and behavioral symptoms were frequent across the overall cohort, with 78.3% of participants meeting the predefined threshold for clinical concern on at least one CBCL scale. Second, no significant differences in CBCL profiles were observed between participants with syndromic and non-syndromic visual impairment, although the relatively small syndromic subgroup limits the interpretation of this finding. Third, participants with mild-to-moderate visual impairment had significantly higher Anxious/Depressed and Somatic Complaints scores than those with severe-to-profound visual impairment, and both differences remained significant after correction for multiple comparisons. Secondary categorical analyses further indicated a higher prevalence of clinically elevated Internalizing Problems, Anxious/Depressed, and Withdrawn/Depressed scores in the mild-to-moderate group.

Before considering subgroup differences, an important observation was the substantial burden of emotional and behavioral symptoms across the entire sample. Nearly 80% of participants presented at least one indicator of scores meeting the predefined thresholds for clinical concern, with internalizing problems, withdrawal and depressive symptoms, and social difficulties being particularly frequent. These findings reinforce growing evidence that childhood visual impairment is associated with significant emotional vulnerability and highlight the importance of incorporating routine psychological assessment into multidisciplinary clinical care, irrespective of the underlying etiology or severity of visual impairment.

### 4.1. Syndromic Status and CBCL Profiles

Contrary to our initial hypothesis, participants with syndromic and non-syndromic visual impairment did not differ significantly in emotional or behavioral symptoms. Previous studies have suggested that co-occurring developmental conditions may contribute substantially to the emotional and behavioral difficulties observed in children with visual impairment [8], while syndrome-specific behavioral phenotypes have also been described in neurogenetic disorders [7]. Our findings did not demonstrate a measurable effect of syndromic status on CBCL profiles in this cohort. Given the small syndromic subgroup (*n* = 14), this comparison was reasonably powered to detect only large-magnitude effects, so the absence of significance carries a high risk of Type II error and should not be read as evidence that syndromic and non-syndromic children have equivalent behavioral profiles; clinically meaningful differences may exist but remain undetected in this cohort. One possible explanation is that visual impairment itself represents a shared developmental challenge across etiologically diverse conditions. Reduced access to visual social cues, limitations in environmental exploration, and altered opportunities for social interaction may constitute common developmental experiences that influence emotional functioning across both syndromic and non-syndromic groups. However, given the relatively small syndromic subgroup, these findings should be interpreted cautiously.

### 4.2. Association Between Visual Impairment Severity and Behavioral Profiles

The main finding of this study was that, contrary to our initial hypothesis, participants with mild-to-moderate visual impairment showed higher Anxious/Depressed and Somatic Complaints scores than those with severe-to-profound visual impairment, with both differences remaining statistically significant after FDR correction. Secondary categorical analyses also identified higher proportions of participants meeting the predefined thresholds for clinical concern for Internalizing Problems, Anxious/Depressed, and Withdrawn/Depressed in the mild-to-moderate group; however, these exploratory comparisons were not adjusted for multiple testing. Taken together, these findings suggest that greater severity of visual impairment is not necessarily accompanied by greater emotional and behavioral symptom burden; however, the present analyses do not establish the shape of the relationship between visual impairment severity and behavioral outcomes.

One possible explanation for this unexpected pattern may involve differences in adaptation to residual visual function. Children with partial vision may encounter situations in which visual information is available but incomplete or unreliable, potentially increasing uncertainty during social interactions, mobility, academic activities, and environmental exploration [13]. By contrast, children with profound visual impairment may develop compensatory strategies adapted to limited or absent visual input from an earlier developmental stage. These possibilities were not directly assessed in the present study and should therefore be regarded as hypotheses for future investigation.

### 4.3. Potential Neurodevelopmental Mechanisms

Beyond psychosocial adaptation, neurodevelopmental mechanisms may also contribute to this pattern. Contemporary neurobiological models of anxiety emphasize uncertainty and unpredictability, rather than solely the objective magnitude of a sensory deficit, as important contributors to sustained threat processing involving limbic and prefrontal circuits [16]. From this perspective, partial visual input could theoretically generate greater perceptual uncertainty than more complete visual deprivation, potentially contributing to increased vulnerability to anxiety-related symptoms. Although this mechanism was not directly assessed in the present study, it provides a neurobiologically plausible framework for interpreting the unexpected pattern observed in our cohort.

Differences in sensory neuroplasticity may also represent a relevant hypothesis. Experimental and clinical evidence indicates that visual deprivation can induce compensatory cross-modal reorganization, with increased recruitment of cortical regions normally involved in visual processing during auditory and tactile tasks [17]. Such neuroplastic adaptations are particularly prominent following early visual deprivation and may support functional compensation in individuals with profound visual loss. In children with residual vision, partial visual input could potentially influence the extent or organization of these compensatory processes, resulting in a different balance between visual dependence and cross-modal adaptation. Whether such differences contribute to emotional or behavioral outcomes remains unknown. Neuroplasticity was not directly assessed in the present study, and this interpretation should therefore be regarded as a mechanistic hypothesis requiring investigation through longitudinal studies incorporating neuroimaging and neurophysiological measures.

### 4.4. Psychosocial Adaptation and the Disability Paradox

Psychosocial factors may also play an important role. The broader disability paradox literature demonstrates that subjective well-being often does not decline proportionally with objective disability severity [18]. Children with residual vision may occupy an ambiguous social position in which their disability is less readily recognized by peers, teachers, and society, potentially reducing access to accommodations and increasing social expectations. Although school placement and rehabilitation history were not assessed in this study, prior literature suggests that disparities in early rehabilitation and educational support may compound this burden. Children with severe-to-profound impairment often receive early specialized interventions, such as mobility training and Braille instruction, whereas those with mild-to-moderate impairment are more frequently mainstreamed into regular classrooms without tailored adaptations [2,9,11,19].

If applicable to the present sample, the continuous effort required to function in predominantly sighted environments, such as straining to read standard print materials, could plausibly contribute to visual fatigue and school-related anxiety. This hypothesis warrants direct investigation in future studies incorporating educational and rehabilitation data. Children with residual vision may also experience greater frustration and social comparison despite having less severe visual impairment. These pressures may be particularly relevant during adolescence, when peer acceptance, social identity, and comparison with sighted peers become increasingly important to psychological well-being [9,19,20,21]. This interpretation is consistent with previous work showing that social inclusion and recognition influence psychosocial adjustment among children and adolescents with visual impairment [19,20], as well as longitudinal evidence indicating that psychological adjustment does not necessarily follow a simple severity-proportional trajectory over time [21].

Previous findings from an overlapping cohort within the same research program showed a different pattern for cognitive and adaptive functioning, with greater visual impairment associated with poorer executive and adaptive outcomes [11]. Together, these observations suggest that different domains of neurodevelopment may relate differently to visual impairment severity. Cognitive and adaptive functioning may be more strongly influenced by reduced access to incidental visual learning, whereas emotional adjustment may additionally reflect perceptual uncertainty, psychosocial adaptation, and compensatory developmental processes.

### 4.5. Limitations

Several limitations should be considered when interpreting these findings. First, the cross-sectional design precludes conclusions regarding causality or developmental trajectories. Second, emotional and behavioral symptoms were assessed exclusively through parent report using the CBCL, which may be subject to informant-related bias. Third, the relatively small syndromic subgroup (*n* = 14) limited statistical power for the etiological comparison and may have reduced the ability to detect smaller between-group differences. Moreover, this subgroup was clinically heterogeneous, comprising distinct genetic and congenital conditions with potentially different neurodevelopmental trajectories and behavioral phenotypes. Grouping these conditions together may therefore have obscured syndrome-specific patterns and precluded meaningful syndrome-specific analyses. Accordingly, the absence of statistically significant differences should not be interpreted as evidence of behavioral equivalence between syndromic and non-syndromic participants.

Fourth, data on potentially important confounding variables, including socioeconomic status, family environment, educational placement, rehabilitation history (e.g., Braille instruction and mobility training), and comorbid neurodevelopmental conditions such as autism spectrum disorder and ADHD, were not available at the individual level for inclusion in multivariable analyses. Regarding socioeconomic status specifically, approximately 70% of participants were treated exclusively through Brazil’s Unified Health System (SUS), which nationally serves an estimated 70% of the population and disproportionately captures lower-income families, as individuals with private insurance largely bypass SUS wait times for specialized care [22]. While this suggests the sample likely skewed toward lower-to-middle socioeconomic strata, individual-level socioeconomic data were not collected, precluding direct characterization or statistical adjustment for this potential confounder. Comorbid autism spectrum disorder, intellectual disability, and ADHD were also not systematically assessed as part of the present behavioral analysis; the inability to account for these and other unmeasured factors limits our ability to isolate the independent associations of syndromic status and visual impairment severity with behavioral outcomes. Fifth, the absence of a typically developing comparison group limits our ability to determine the extent to which the observed emotional and behavioral profiles are specific to children and adolescents with visual impairment.

Finally, although FDR correction reduced the likelihood of false-positive findings in the primary analyses, it may also have reduced sensitivity to smaller effects. In addition, the secondary categorical analyses were exploratory and were not adjusted for multiple comparisons; these findings should therefore be interpreted cautiously and require replication in larger independent samples.

### 4.6. Clinical and Research Implications

These findings emphasize that emotional symptoms should not be viewed solely as a consequence of profound visual loss. Clinicians should recognize that children with residual vision may also experience substantial emotional and behavioral difficulties that could be underestimated because of their greater functional vision. Routine psychological screening should therefore be considered across the spectrum of pediatric visual impairment, regardless of syndromic status or severity.

Future longitudinal studies involving larger and more deeply phenotyped cohorts should investigate how visual impairment severity interacts with adaptive functioning, syndromic etiology, neurodevelopmental comorbidities, educational environment, and rehabilitation history to shape emotional and behavioral outcomes throughout childhood and adolescence. Studies incorporating neuroimaging and neurophysiological measures may also help determine whether differences in cross-modal plasticity, perceptual uncertainty, and compensatory neural processes contribute to the behavioral patterns observed in the present study.

## 5. Conclusions

In this study of Brazilian children and adolescents with visual impairment, no significant differences in emotional or behavioral symptoms were observed according to syndromic status, although this finding should be interpreted cautiously given the limited size of the syndromic subgroup. In contrast, participants with mild-to-moderate visual impairment showed significantly higher Anxious/Depressed and Somatic Complaints scores than those with severe-to-profound impairment, with both differences remaining significant after FDR correction. These findings suggest that emotional adjustment in pediatric visual impairment may not vary proportionally with the degree of visual loss and that children with residual vision may have emotional and behavioral needs that are not readily recognized.

More broadly, the high proportion of participants meeting predefined thresholds for clinical concern across the overall cohort highlights the importance of emotional and behavioral assessment in children and adolescents with visual impairment. Routine psychological screening should therefore be considered as part of multidisciplinary care across the spectrum of pediatric visual impairment. Future longitudinal studies involving larger samples should clarify how visual impairment severity, adaptive functioning, syndromic etiology, co-occurring neurodevelopmental conditions, educational environment, and rehabilitation history interact with emotional and behavioral outcomes throughout development.

## Figures and Tables

**Table 1:** Demographic characteristics by group.

Group	*N*	Age, Median (Years, IQR)	Male, *n* (%)	Female, *n* (%)	*p*
**Syndromic vs. Non-Syndromic**					
Syndromic	14	11.5 (9.0–13.8)	6 (43%)	8 (57%)	0.559
Non-Syndromic	55	11.0 (7.0–14.0)	29 (53%)	26 (47%)	
**Visual Impairment Severity**					
Mild-to-moderate	33	12.0 (8.0–15.0)	19 (58%)	14 (42%)	0.464
Severe-to-profound	36	11.0 (8.0–14.0)	16 (44%)	20 (56%)	

IQR: interquartile range. Visual impairment severity distribution by etiology was as follows: syndromic, 9 mild-to-moderate and 5 severe-to-profound; non-syndromic, 24 mild-to-moderate and 31 severe-to-profound. Bold text indicates subgroup classification headings.

**Table 2:** CBCL T-scores and proportion of participants meeting the predefined thresholds for clinical concern across the full analytic sample (*N* = 69).

CBCL Scale	Median T-Score (IQR)	*n* (%) in Clinical Range
**Composite Domains (T ≥ 65)**		
Total Problems	64 (59–71)	33 (47.8%)
Internalizing Problems	65 (57–71)	33 (47.8%)
Externalizing Problems	58 (51–66)	21 (30.4%)
**Behavioral Profile (T ≥ 70)**		
Anxious/Depressed	57 (52–66)	11 (15.9%)
Withdrawn/Depressed	67 (57–77)	30 (43.5%)
Somatic Complaints	61 (56–66)	11 (15.9%)
Social Problems	68 (59–73)	28 (40.6%)
Thought Problems	64 (54–70)	21 (30.4%)
Attention Problems	64 (56–68)	13 (18.8%)
Rule-Breaking Behavior	53 (51–57)	7 (10.1%)
Aggressive Behavior	58 (52–68)	12 (17.4%)
**DSM-Oriented Scales (T ≥ 70)**		
Depressive Problems	65 (59–72)	22 (31.9%)
Anxiety Problems	61 (55–66)	14 (20.3%)
Somatic Problems	54 (50–61)	4 (5.8%)
ADHD Problems	62 (54–69)	14 (20.3%)
Oppositional Defiant Problems	55 (51–63)	9 (13.0%)
Conduct Problems	54 (50–61)	9 (13.0%)

Data are presented as median T-score (IQR) and *n* (%). Thresholds for clinical concern were defined as T ≥ 65 for broadband composite scales and T ≥ 70 for syndrome and DSM-oriented scales, according to ASEBA guidelines [3]. IQR, interquartile range; CBCL, Child Behavior Checklist. Bold text indicates subgroup classification headings.

**Table 3:** Proportion of participants meeting the predefined thresholds for clinical concern according to visual impairment severity.

CBCL Scale	Mild-to-Moderate (*N* = 33); *n* (%)	Severe-to-Profound (*N* = 36); *n* (%)	*p*
	**T ≥ 65**	**T ≥ 65**	
Total Problems	19 (57.6%)	12 (33.3%)	0.098
Internalizing Problems	25 (75.8%)	11 (30.6%)	<0.001 *
Externalizing Problems	11 (33.3%)	8 (22.2%)	0.197
	**T ≥ 70**	**T ≥ 70**	
Anxious/Depressed	9 (27.3%)	2 (5.6%)	0.022 *
Withdrawn/Depressed	20 (60.6%)	10 (27.8%)	0.005 *

Note: Thresholds for clinical concern were defined as T ≥ 65 for broadband composite scales and T ≥ 70 for syndrome scales, according to ASEBA guidelines [3]. Bold text indicates the clinical threshold applicable to each group of CBCL scales. *p*-values were calculated using Fisher’s exact test, with *p* < 0.05 considered statistically significant. * indicates statistical significance at *p* < 0.05. These categorical comparisons were conducted as secondary exploratory analyses and were not adjusted for multiple comparisons.

## Data Availability

The data presented in this study are available on request from the corresponding author, because the data are not publicly available due to privacy or ethical restrictions.

## References

[1] Du K., Patil P., Boland M.V. (2025). Mental health of US children with vision impairment: An analysis of the National Health Interview Survey. Am. J. Ophthalmol..

[2] Li D., Chan V.F., Virgili G., Piyasena P., Negash H., Whitestone N., O’Connor S., Xiao B., Clarke M., Cherwek D.H. (2022). Impact of vision impairment and ocular morbidity and their treatment on depression and anxiety in children: A systematic review. Ophthalmology.

[3] Achenbach T.M., Rescorla L.A. (2001). Manual for the ASEBA School-Age Forms & Profiles.

[4] Wang X., Hong X., Cui Y., Li Y. (2024). Revisiting the 8-syndrome of the Child Behavior Checklist (CBCL) in 67,257 Chinese school students. Crit. Public Health.

[5] American Psychiatric Association (2013). Diagnostic and Statistical Manual of Mental Disorders.

[6] Samhan S.M., Alqasim S.A., Aldwsri A.S., Alhazmi M.A., Azzoni R.I., Mugharbel L.H., Hazmi S.M., Almohaish R.A., Zaebi S.H., Alghalyini B. (2025). A Comprehensive Review of Congenital Eye Diseases in Pediatrics: Etiology, Diagnosis, and Management. Cureus.

[7] Hodapp R.M., Dykens E.M. (2005). Measuring behavior in genetic disorders of mental retardation. Ment. Retard. Dev. Disabil. Res. Rev..

[8] Alimović S. (2013). Emotional and behavioural problems in children with visual impairment, intellectual and multiple disabilities. J. Intellect. Disabil. Res..

[9] Cheng Y., Wang S., Pavlopoulou G., Hayton J., Sideropoulos V. (2025). Mental Health and Quality of Life in Children and Young People with Vision Impairment: A Systematic Review. Neurodiversity.

[10] Ammerman R.T., Van Hasselt V.B., Hersen M. (1986). Psychological adjustment of visually handicapped children and youth. Clin. Psychol. Rev..

[11] Correia K., Bara T.S., Furlin V., Prando C., Iankilevich P.G., Cordeiro M.L. (2026). Impact of visual impairment severity on working memory and executive function in children and adolescents. Front. Psychol..

[12] Feng J., Garcia-Piña F., Beheshti M., Hudson T.E., Seiple W., Rizzo J.R. (2026). Residual gaze behaviour during navigation in blindness and low vision. Disabil. Rehabil. Assist. Technol..

[13] Dandona L., Dandona R. (2006). Revision of visual impairment definitions in the International Statistical Classification of Diseases. BMC Med..

[14] Bordin I.A.S., Mari J.J., Caeiro M.F. (1995). Validação da versão brasileira do “Child Behavior Checklist” (CBCL) (Inventário de Comportamentos da Infância e Adolescência): Dados preliminares. Rev. ABP-APAL.

[15] Bordin I.A., Rocha M.M., Paula C.S., Teixeira M.C.T.V., Achenbach T.M., Rescorla L.A., Silvares E.F.M. (2013). Child Behavior Checklist (CBCL), Youth Self-Report (YSR) and Teacher’s Report Form (TRF): An overview of the development of the original and Brazilian versions. Cad. Saúde Pública.

[16] Grupe D.W., Nitschke J.B. (2013). Uncertainty and anticipation in anxiety: An integrated neurobiological and psychological perspective. Nat. Rev. Neurosci..

[17] Théoret H., Merabet L., Pascual-Leone A. (2004). Behavioral and neuroplastic changes in the blind: Evidence for functionally relevant cross-modal interactions. J. Physiol. Paris.

[18] Albrecht G.L., Devlieger P.J. (1999). The disability paradox: High quality of life against all odds. Soc. Sci. Med..

[19] Manitsa I., Barlow-Brown F., Livanou M. (2024). Evaluating the role of social inclusion in the self-esteem and academic inclusion of adolescents with vision impairment. Br. J. Vis. Impair..

[20] Adhikari S., Elsman E.B.M., van Nispen R.M.A., Van Rens F., Poudel M., van Rens G.H.M.B. (2025). Participation and quality of life of Nepalese children with visual impairment in comparison with normally sighted peers: A cross-sectional comparative study. J. Patient-Rep. Outcomes.

[21] Pinquart M., Pfeiffer J.P. (2014). Change in psychological problems of adolescents with and without visual impairment. Eur. Child Adolesc. Psychiatry.

[22] Vieira Y.P., Rocha J.Q.S., Dutra R.P., Nunes L.S., Duro S.M.S., Saes M.O. (2023). Socioeconomic inequities in specialized health services use following COVID-19 in individuals from Southern Brazil. BMC Health Serv. Res..

